# Parental emotional warmth and career choice anxiety: a chain-mediation study in China

**DOI:** 10.3389/fpsyg.2026.1813789

**Published:** 2026-05-21

**Authors:** Meng-Te Hung, Jingyi Wu, Li-Ching Hung, Cary Stacy Smith, Ling-Shen Hung

**Affiliations:** 1 School of Liberal Arts, Minnan Normal University, Zhangzhou, China; 2 School of Cross-Border E-Commerce, Yango University, Fuzhou, China; 3 Department of Applied Psychology, Yango University, Fuzhou, China; 4 School of Food and Bioengineering, Fujian Polytechnic Normal University, Fuzhou, China

**Keywords:** career choice anxiety, chain mediation, Chinese college students, life satisfaction, parental emotional warmth, psychological resilience

## Abstract

Career choice anxiety is a growing concern among Chinese university students and may be shaped by family expectations and labor-market uncertainty. This study examined whether the association between parental emotional warmth and career choice anxiety was indirectly linked through life satisfaction and psychological resilience. A total of 590 students completed validated questionnaires; after screening, 508 father and 560 mother reports were retained for analysis. Structural equation modeling indicated that parental emotional warmth was associated with lower career choice anxiety, and this association showed significant indirect effects through life satisfaction and psychological resilience, both independently and sequentially. No significant differences were found between paternal and maternal emotional warmth. These findings are statistically consistent with the proposed mediation model and highlight the potential relevance of family-related emotional support in understanding career choice anxiety among Chinese university students.

## Introduction

Career choice anxiety refers to feelings of tension, unease, worry, and persistent emotional distress experienced by individuals, particularly first-time job-seeking university graduates, when confronted with occupational choices and career decisions, often accompanied by physiological and behavioral reactions (Zhang and Chen, 2006). In recent years, with the continuous expansion of university enrollment and the persistent growth in the number of graduates, employment competition among university students has become increasingly fierce. It is projected that the class of 2025 will include 12.22 million university graduates, an increase of 430,000 year-on-year and a new record high (Guo and Li, 2025). Against this backdrop, career choice anxiety has become increasingly prevalent among university students and has emerged as an important issue affecting both mental health and career adaptability (Zhang and Yao, 2011). Moreover, it is closely associated with social phenomena such as “slow employment,” making it a pressing concern in the transition from higher education to the labor market.

Although career choice anxiety is often discussed as a general developmental issue, career decision-making is not culturally uniform. Cross-cultural evidence has shown that young people differ in their career decision-making profiles and difficulties across national contexts, reflecting the influence of cultural values, family expectations, and social norms on career-related choices (Willner et al., 2015). In the Chinese context, career choice is often shaped not only by individual interests and abilities but also by family expectations, relational obligations, and labor-market uncertainty. Thus, career choice anxiety among Chinese university students should be understood not merely as an individual emotional response, but also as a culturally embedded developmental challenge.

Among the many factors influencing career choice anxiety, the family environment—particularly parental emotional warmth—has received increasing attention. Parental emotional warmth is widely recognized as a positive parenting style that is associated with psychological adjustment and lower levels of maladaptive emotional outcomes (Collins and Laursen, 2004; Repetti et al., 2002). Recent studies have also shown that parental emotional warmth is associated with greater proactive career exploration among university students and lower levels of negative emotions such as anxiety and depression (Maftei et al., 2022). This issue may be especially important in China, where parents are often deeply involved in children’s educational and career development, and where career decisions are commonly negotiated within a family framework. However, research specifically examining how parental emotional warmth is associated with career choice anxiety remains limited. Existing studies have often focused on single mediators, such as self-efficacy (Xing et al., 2023) or career cognitions (e.g., career decision-making clarity) (Doğanülkü et al., 2026), while fewer studies have integrated multiple psychological mechanisms into a single explanatory model.

Recent research on Chinese students suggests that career-related adjustment is shaped by both social support and internal psychological resources. For example, career decision-making has been linked to variables such as career adaptability and educational identity (Wang et al., 2025), and social support has been shown to influence career decision-making difficulties indirectly through psychological capital and career decision-making self-efficacy (Zhou et al., 2024). These findings indicate that career-related anxiety in Chinese students may be better understood through mechanisms that connect family context with broader psychological adjustment. In this regard, life satisfaction and psychological resilience may be particularly important. Life satisfaction, as the core cognitive component of subjective well-being, reflects an individual’s overall evaluation of life quality (Jia et al., 2021), whereas psychological resilience refers to the capacity to maintain or restore adaptive functioning in the face of adversity (Fletcher and Sarkar, 2013). Both variables have been shown to be closely related to anxiety reduction and adaptive coping (Diener et al., 2018; Southwick and Charney, 2012).

Although existing studies have separately established the importance of parental emotional warmth, life satisfaction, and psychological resilience in adolescent and university students’ psychological adaptation (Nie et al., 2025; Wang et al., 2022; Müceldili et al., 2023), research systematically integrating these factors within the context of career choice anxiety remains scarce. In particular, little is known about whether parental emotional warmth is associated with career choice anxiety through the serial psychological mechanisms of life satisfaction and psychological resilience in the Chinese context. To address this gap, the present study proposes a multidimensional serial mediation model grounded in Self-Determination Theory and Ecological Systems Theory. Specifically, we examine whether parental emotional warmth is associated with lower career choice anxiety through life satisfaction and psychological resilience. By clarifying these pathways, this study seeks to deepen understanding of career-related emotional adjustment among Chinese university students and to provide implications for family education, psychological intervention, and career counseling.

## Literature review

### Parental emotional warmth and career anxiety

The factors contributing to career choice anxiety among university students include both individual factors, such as career expectations and self-management (Wang et al., 2022), and external environmental factors, such as family, school, and social support (Yang et al., 2021a,b). Grounded in Ecological Systems Theory (Bronfenbrenner, 1986), the family is recognized as a critical system shaping psychological and behavioral development. A warm and supportive family environment may facilitate better coping with external challenges (Rosa and Tudge, 2013). Parental emotional warmth, as a positive parenting style, refers to parents’ attentiveness to children’s emotional needs through care, responsiveness, and support during interactions (Baumrind, 1991). Extensive research indicates that parental emotional warmth is associated with a sense of security and positive psychological resources, such as hope, optimism, resilience, and self-efficacy, thereby potentially helping university students face career decision-making pressures with greater confidence and composure (Hou et al., 2013).

Cross-cultural research has shown that career decision-making difficulties are shaped by culturally specific norms and expectations rather than representing a culturally neutral developmental issue (Willner et al., 2015). This perspective is particularly relevant when examining parental emotional warmth in China. In collectivistic cultures, high parental involvement is often interpreted as care and responsibility, whereas similar behaviors may be viewed as interference in more individualistic contexts (Rudy and Grusec, 2006; Lorenzo-Blanco et al., 2012). Chinese parents may also express support through encouraging expectations, but when such expectations exceed children’s perceived capacities, they may become a source of pressure and anxiety (Zhou et al., 2025). Accordingly, parental emotional warmth may play a particularly important role in shaping how Chinese university students experience and cope with career uncertainty. However, the specific mechanism through which parental emotional warmth is associated with career choice anxiety remains insufficiently understood.

### Life satisfaction as a mediator

Current literature seldom directly examines the relationship among parental emotional warmth, life satisfaction, and career choice anxiety. Nevertheless, previous studies have separately demonstrated that parental warmth is positively associated with life satisfaction and that life satisfaction is negatively associated with anxiety. Research indicates that parenting styles are significantly associated with life satisfaction, with emotional warmth showing a robust positive association (Liu et al., 2020). From the perspective of Self-Determination Theory (Ryan and Deci, 2000), parental emotional warmth may be associated with higher subjective well-being by satisfying basic psychological needs for autonomy, competence, and relatedness (Chirkov and Ryan, 2021).

Life satisfaction has also been linked to both psychological well-being and career development among university students (Ji et al., 2024). Because this group faces multiple sources of pressure, including academic, interpersonal, and career-related stress, life satisfaction may serve as a cognitive-emotional buffer that is associated with a less threatening appraisal of career uncertainty (Zhang and Fan, 2021; Müceldili et al., 2023). In addition, recent research on Chinese students has shown that career decision-making is closely associated with internal psychological resources, such as career adaptability and educational identity (Wang et al., 2025). This suggests that life satisfaction may represent an important pathway through which supportive family experiences are translated into more adaptive career-related emotional outcomes. Nevertheless, the mediating role of life satisfaction in the relationship between parental emotional warmth and career choice anxiety has not yet been systematically verified.

### Psychological resilience as a mediator

Rooted in attachment theory, the emotional bond formed between children and caregivers has lasting implications for psychological adaptation across development (Maxwell et al., 2013). Individuals with secure attachment typically perceive greater support and cope more adaptively with stress, whereas those with insecure attachment may show maladaptive adjustment patterns (Feeney and Collins, 2019). Children raised in emotionally warm family environments are more likely to develop psychological security and belongingness, which may later be internalized as coping resources. Empirical studies have shown that parental emotional warmth is significantly associated with higher psychological well-being and resilience in children and adolescents (Deng and Luo, 2024; Wang et al., 2024).

Psychological resilience refers to the capacity to maintain positive adaptation in the face of adversity (Yu and Zhang, 2005). Individuals with higher resilience generally demonstrate stronger coping abilities, greater intrinsic motivation, and lower anxiety under stressful conditions (Wang et al., 2022). Although some studies suggest that high resilience may occasionally be associated with overanalysis in career decision-making contexts (Kenner and Sage, 2023), the broader literature more consistently supports its protective role. In addition, growing evidence indicates that resilience is involved in the association between parental warmth and anxiety-related outcomes (Zhang and Li, 2024; Huang and Jiang, 2024). Yang et al. (2021a,b) further found that university students who perceive greater parental emotional warmth tend to adopt more proactive and self-assured strategies when facing career stressors. Taken together, these findings suggest that psychological resilience may be an important mechanism linking parental emotional warmth to lower career choice anxiety, although this pathway still requires direct empirical examination.

### Chain mediation mechanism

Building upon prior research, life satisfaction and psychological resilience may jointly function as serial mediators linking parental emotional warmth to career choice anxiety. A key theoretical issue in this sequential pathway concerns why life satisfaction is positioned before psychological resilience. In the present study, life satisfaction is conceptualized as a broader cognitive-evaluative appraisal of one’s overall life circumstances, whereas psychological resilience is understood as a more proximal adaptive capacity that may help individuals cope effectively with stress and uncertainty. From this perspective, parental emotional warmth may first be associated with students’ stronger overall sense of life quality, security, and personal worth, which then provides a positive psychological foundation for the development or activation of resilience when career-related pressures arise.

This ordering is consistent with the Broaden-and-Build Theory (Fredrickson, 1998), which proposes that positive emotional and cognitive states broaden individuals’ thought–action repertoires and gradually build enduring personal resources. In this sense, higher life satisfaction may help students interpret challenges in a less threatening manner, maintain a more optimistic orientation toward the future, and develop stronger confidence in their ability to withstand setbacks. These positive evaluations may, in turn, foster psychological resilience, which serves as a more direct protective resource in the face of career uncertainty and anxiety. Likewise, Luthar et al. (2000) emphasized that resilience is not a fixed trait but a dynamic adaptation process shaped by supportive environments and constructive appraisals. Thus, life satisfaction may reasonably precede resilience in the present model because it reflects a more general positive evaluation of life, whereas resilience reflects the capacity to mobilize adaptive responses under stress.

This proposition is also consistent with recent findings from Chinese samples showing that support-related factors influence career decision-making difficulties indirectly through sequential psychological mechanisms. Specifically, Zhou et al. (2024) found that social support was associated with lower career decision-making difficulties through the chain-mediating roles of psychological capital and career decision-making self-efficacy. Although their study focused on different mediators, it provides important contextual support for the view that, among Chinese students, support-related variables may operate through internal psychological resources rather than through direct effects alone.

**Figure 1:**
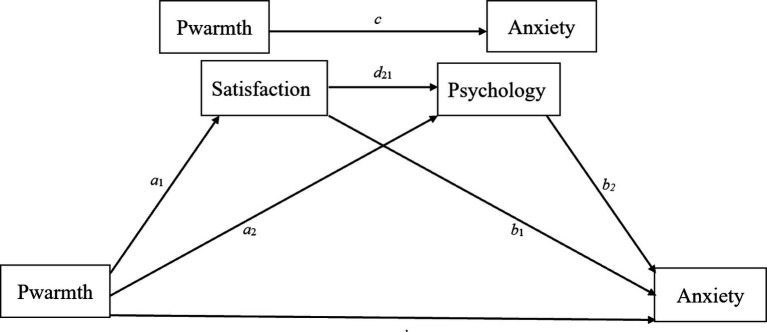
The statistical model in which the effect of parental emotional warmth on career choice anxiety is mediated by life satisfaction and psychological resilience. Pwarmth = parental emotional warmth; Satisfaction = life satisfaction; Psychology = psychological resilience; Anxiety = career choice anxiety.

In summary, although previous studies have examined the relationships among parental emotional warmth, life satisfaction, psychological resilience, and anxiety from separate perspectives, empirical research integrating these variables within a unified framework of career choice anxiety remains limited. Moreover, insufficient attention has been paid to how these mechanisms operate in the Chinese context, where family involvement and career-related pressures are often closely intertwined. Grounded in Self-Determination Theory and Ecological Systems Theory, the present study proposes a serial mediation model in which parental emotional warmth is associated with career choice anxiety through life satisfaction and psychological resilience (see Figure 1). Accordingly, the following hypotheses are proposed:

*H1*: Life satisfaction mediates the relationship between parental emotional warmth and career choice anxiety among college students.

*H2*: Psychological resilience mediates the relationship between parental emotional warmth and career choice anxiety among college students.

*H3*: Life satisfaction and psychological resilience serve as serial mediators between parental emotional warmth and career choice anxiety among college students.

## Method

### Participants

This study targeted Chinese college students in Southeast China to examine the relationships among perceived parental emotional warmth, psychological resilience, life satisfaction, and career choice anxiety. Data were collected via the online platform “Wenjuanxing” using a purposive sampling method (Fraenkel et al., 2019). A total of 590 responses were received. After data screening and separating responses by parental figure, 508 valid cases were retained for the father sample and 560 for the mother sample. In father sample: among the 508 respondents, 99 (19.5%) were male and 409 (80.5%) were female. Most participants came from dual-parent families (83.5%), while 5.3% came from single-parent families, and 11.2% from other family structures. Grade levels ranged from freshman to graduate students, with ages between 18 and 28 years (*M* = 20.82, *SD* = 1.679). In mother sample: among the 560 respondents, 105 (18.8%) were male and 455 (81.3%) were female. Participants from dual-parent families accounted for 74.6%, those from single-parent families for 13.1%, and other family types for 12.3%. Ages ranged from 18 to 28 years (*M* = 20.81, *SD* = 1.735).

### Measures

#### Parental emotional warmth

Perceived parental emotional warmth was measured using the Chinese short version of the s-EMBU (Egna Minnen av. Barndoms Uppfostran), originally developed by Perris et al. (1980) and revised by Jiang et al. (2010). The scale consists of 21 items covering three dimensions: Rejection (6 items), Emotional Warmth (7 items), and Overprotection (8 items), scored on a 6-point Likert scale (1 = *never*, 6 = *always*). Item 15 was reverse-coded. Higher scores indicate that participants perceive higher levels of parental emotional warmth.

**Figure 2:**
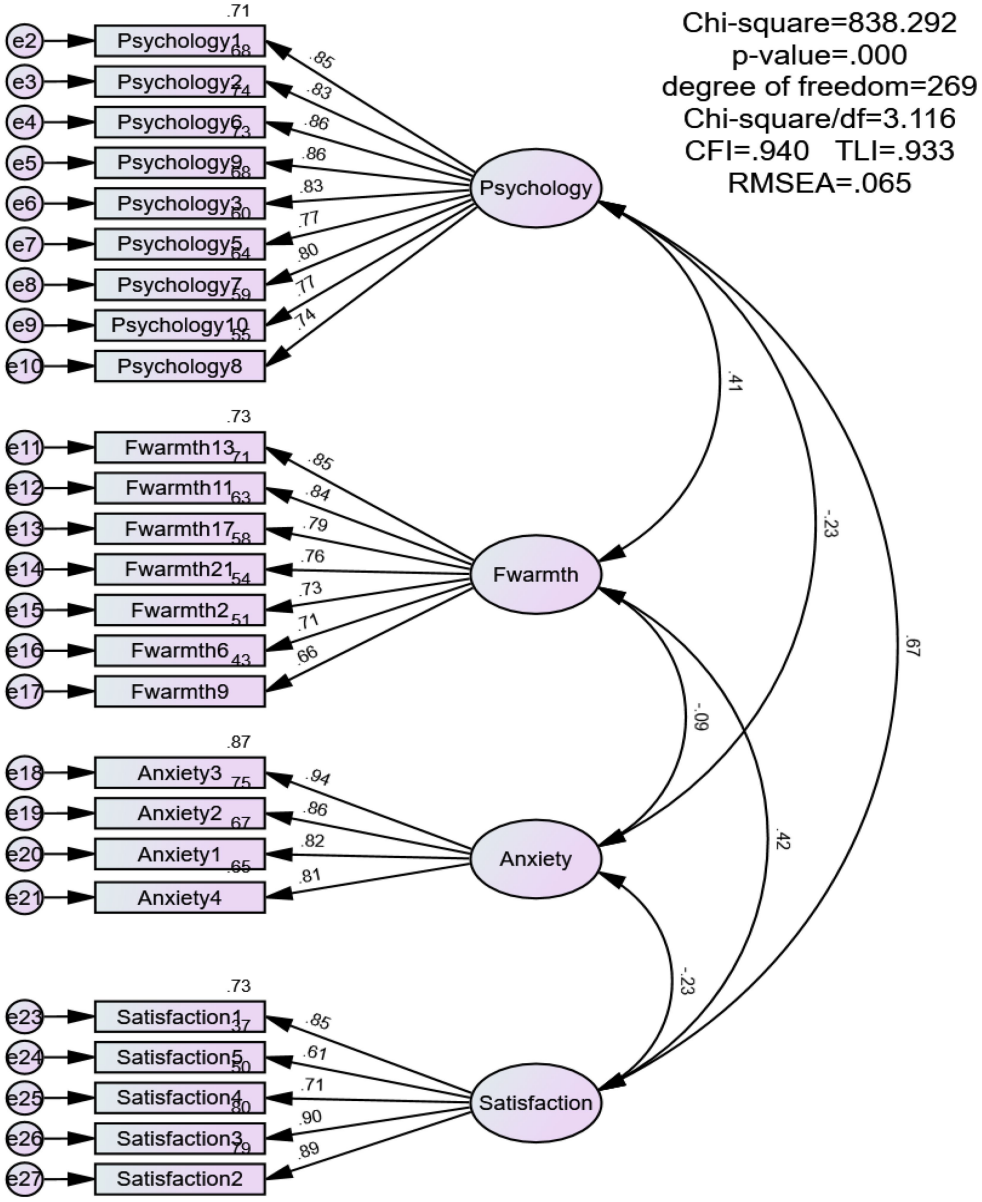
The CFA model summary information (*N =* 508). Fwarmth = father’s emotional warmth; Satisfaction = life satisfaction; Psychology = psychological resilience; Anxiety = career choice anxiety.

**Figure 3:**
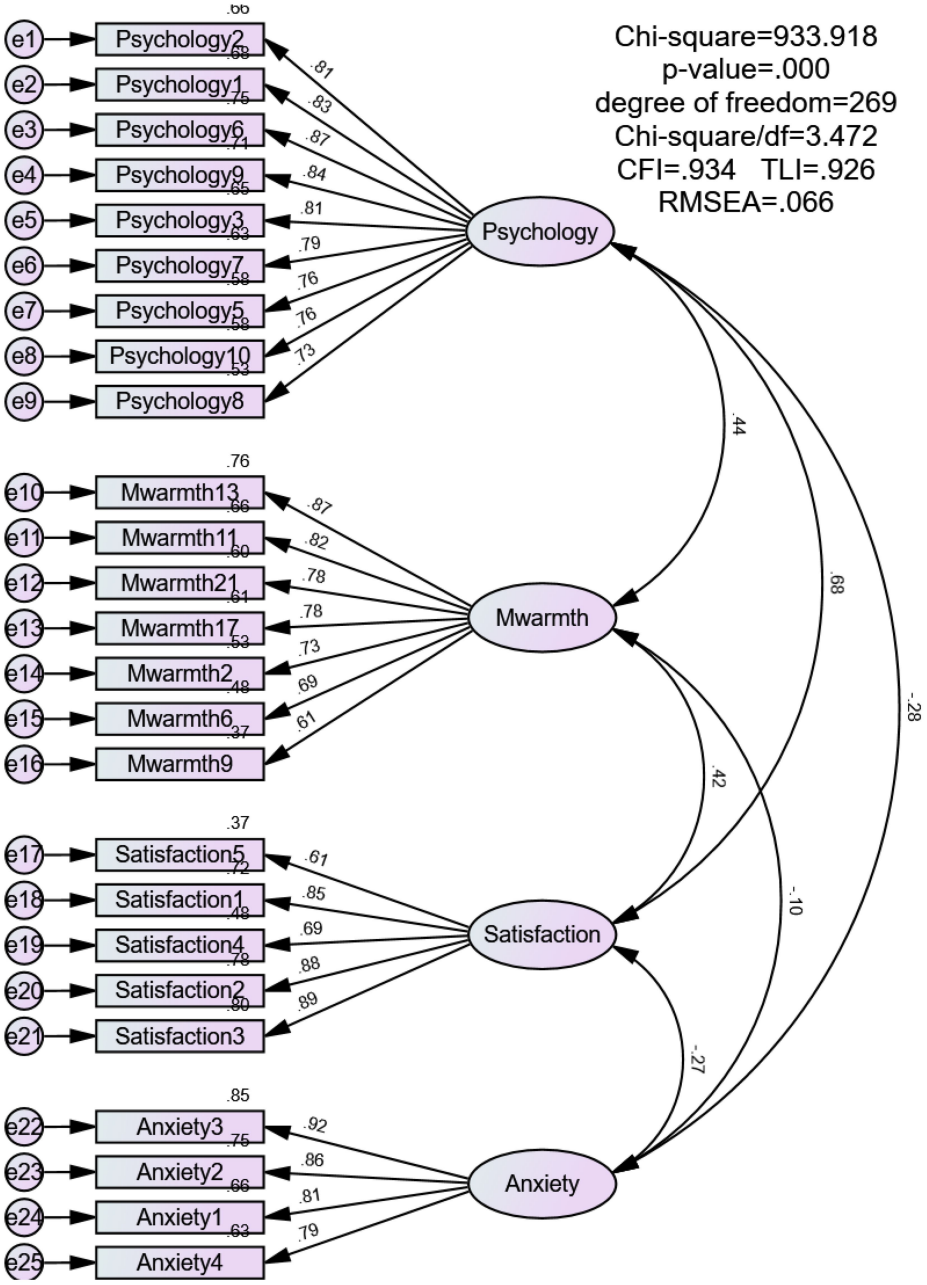
The CFA model summary information (*N =* 560). Mwarmth = mother’s emotional warmth; Satisfaction = life satisfaction; Psychology = psychological resilience; Anxiety = career choice anxiety.

Only the Emotional Warmth dimension was used for this study. A confirmatory factor analysis (CFA; Hair et al., 2010) confirmed good factor structure with all 7 items retained (see Figures 2, 3). Cronbach’s α values were 0.908 (father sample) and 0.901 (mother sample), indicating excellent reliability (see Tables 1, 2).

**Table 1:** Summary of the reliability and validity analysis for study variables (*N* = 508).

Measure	α (ω)	CR	AVE	Pearson correlations			
				1	2	3	4
1. Psychology	0.943 (0.943)	0.945	0.659	**0.812**			
2. Fwarmth	0.908 (0.909)	0.909	0.588	0.409***	**0.767**		
3. Anxiety	0.915 (0.914)	0.918	0.738	−0.231***	−0.092	**0.859**	
4. Satisfaction	0.889 (0.888)	0.896	0.638	0.673***	0.421***	−0.231***	**0.799**

Fwarmth = father’s emotional warmth; Satisfaction = life satisfaction; Psychology = psychological resilience; Anxiety = career choice anxiety. ***p < 0.001; **p < 0.01; *p < 0.05. α = Cronbach’s alpha (α) coefficient; ω = McDonald’s omega (ω) coefficient. CR = composite reliability; AVE = average variance extracted. The square roots of the AVEs appear in bold on the diagonal. Correlations are shown below the diagonal.

**Table 2:** Summary of the reliability and validity analysis for study variables (*N* = 560).

Measure	α (ω)	CR	AVE	Pearson correlations			
				1	2	3	4
1. Psychology	0.939 (0.939)	0.941	0.642	**0.801**			
2. Mwarmth	0.901 (0.903)	0.903	0.573	0.437***	**0.757**		
3. Satisfaction	0.885 (0.883)	0.893	0.630	0.680***	0.421***	**0.793**	
4. Anxiety	0.908 (0.907)	0.912	0.722	−0.275***	−0.098*	−0.274***	**0.850**

Mwarmth = mother’s emotional warmth; Satisfaction = life satisfaction; Psychology = psychological resilience; Anxiety = career choice anxiety. ***p < 0.001; **p < 0.01; *p < 0.05. α = Cronbach’s alpha (α) coefficient; ω = McDonald’s omega (ω) coefficient. CR = composite reliability; AVE = average variance extracted. The square roots of the AVEs appear in bold on the diagonal. Correlations are shown below the diagonal.

#### Career choice anxiety

Career choice anxiety was assessed using a 5-item adapted version of the Self-Rating Anxiety Scale (SAS; Zung, 1971), revised by Wang and Chi (1984). One item (CCA5) was removed after CFA because it showed comparatively weak standardized factor loadings in both the father sample (0.42) and the mother sample (0.43), both below the commonly used 0.50 criterion for standardized loadings (Hair et al., 2010), and was less directly aligned with career choice anxiety in the present context. The retained four items continued to capture the core affective features of career choice anxiety. Detailed item-level loadings before and after item removal, together with the original Chinese item wording, are reported in the Supplementary Tables S1, S2 and Supplementary Figures S1, S2. The final 4-item scale (see Figures 2, 3) was scored on a 7-point Likert scale (1 = *strongly disagree*, 7 = *strongly agree*). Higher scores indicated greater anxiety related to future employment. Cronbach’s *α* values were 0.915 for the father sample and 0.908 for the mother sample, indicating strong reliability (see Tables 1, 2).

#### Life satisfaction

Life satisfaction was measured using the Satisfaction with Life Scale (SWLS) by Diener et al. (1985), adapted into Chinese by Qiu et al. (2008). The 5-item unidimensional scale uses a 7-point Likert format (1 = *Strongly Disagree*, 7 = *Strongly Agree*). Higher total scores indicate greater life satisfaction. All items were retained after CFA (see Figures 2, 3). Cronbach’s α were 0.889 (father sample) and 0.885 (mother sample), indicating high internal consistency (see Tables 1, 2).

#### Psychological resilience

Psychological resilience was assessed with the 10-item Chinese version of the CD-RISC-10, developed by Campbell-Sills and Stein (2007) and validated by Yu and Zhang (2007). One item (PR4) was removed after CFA because it showed comparatively weak standardized factor loadings in both the father sample (0.43) and the mother sample (0.47), both below the commonly used 0.50 criterion for standardized loadings (Hair et al., 2010), and contributed less strongly to construct coherence in the present sample. The retained nine items continued to represent the core conceptual domain of psychological resilience. Detailed item-level loadings before and after item removal, together with the original Chinese item wording, are reported in the Supplementary Tables S3, S4 and Supplementary Figures S1, S2. The final 9-item scale (see Figures 2, 3) was scored on a 7-point Likert scale (1 = *strongly disagree*, 7 = *strongly agree*). Higher total scores indicated greater psychological resilience. Cronbach’s α values were 0.943 for the father sample and 0.939 for the mother sample, indicating excellent reliability (see Tables 1, 2).

### Procedure

Data for this study were collected from March to May 2025 using the Wenjuanxing online survey platform, which is widely used for academic research in China. The target population was university students enrolled in higher education institutions in southeastern China. A non-probability purposive sampling method was employed. The survey link was distributed through university-based online forums, academic WeChat groups, and email lists, allowing voluntary and anonymous participation.

Participants were first provided with an informed consent statement outlining the study’s purpose, the confidentiality of responses, the voluntary nature of participation, and their right to withdraw at any time without penalty. Only those who confirmed their consent were allowed to proceed with the questionnaire.

The online questionnaire consisted of four standardized self-report instruments measuring parental emotional warmth, life satisfaction, psychological resilience, and career choice anxiety. To improve data quality, attention-check items and logic constraints were embedded. After data screening, a total of 508 valid responses for the father sample and 560 for the mother sample were retained for analysis.

This study was approved by the university’s institutional ethics committee. All participants provided informed consent prior to participation. All procedures were conducted in accordance with the ethical standards of the Declaration of Helsinki.

### Data analysis

This study adopted multiple statistical analysis methods to explore the complex relationships among the independent variable—parental emotional warmth—and the dependent variables—college students’ career choice anxiety, life satisfaction, and psychological resilience. SPSS 27.0 and Amos 26.0 software were used to conduct descriptive analysis, correlation analysis, path analysis, and mediation effect testing. Finally, research results, discussion, and suggestions were written based on the statistical analysis.

To ensure the adequacy of the mediation model, various goodness-of-fit indices were used to evaluate whether the model had good fitness, including chi-square value (χ^2^)/degrees of freedom (df), Tucker–Lewis Index (TLI), Comparative Fit Index (CFI), Standardized Root Mean Square Residual (SRMR), and Root Mean Square Error of Approximation (RMSEA).

## Results

### Common method Bias analysis

To assess the potential influence of common method bias (CMB), both procedural and statistical remedies were applied. Procedurally, participants were assured of anonymity and informed that there were no right or wrong answers, which may help reduce evaluation apprehension. Statistically, Harman’s single-factor test was first conducted (Podsakoff et al., 2003). In both the father-report sample and the mother-report sample, four factors had eigenvalues greater than 1, and the first factor accounted for 38.813% and 38.752% of the total variance, respectively, both below the recommended 40% threshold.

Because Harman’s test is generally regarded as a relatively coarse diagnostic and may lack sensitivity in detecting method effects, an additional CFA-based comparison was conducted to provide a more stringent assessment. In the father sample, the single-factor model showed poor fit to the data, χ^2^ = 4709.289, df = 275, CFI = 0.531, RMSEA = 0.178, whereas the hypothesized multifactor measurement model showed substantially better fit, χ^2^ = 838.292, df = 269, CFI = 0.940, RMSEA = 0.065. Similarly, in the mother sample, the single-factor model also demonstrated poor fit, χ^2^ = 4878.500, df = 275, CFI = 0.541, RMSEA = 0.173, whereas the hypothesized multifactor measurement model fit the data much better, χ^2^ = 933.918, df = 269, CFI = 0.934, RMSEA = 0.066. Taken together, these results suggest that CMB is less likely to fully account for the observed associations among the study variables, although shared method variance cannot be ruled out entirely.

### Model fit analysis

This study tested the measurement model fit for the four variables: parental emotional warmth, college students’ career choice anxiety, life satisfaction, and psychological resilience. In the father sample, the overall model’s χ^2^/df fell between 1 and 5, meeting the standard; CFI = 0.940 (greater than 0.90), SRMR = 0.042 (less than 0.08), RMSEA = 0.065 (less than 0.08). In the mother sample, χ^2^/df also fell between 1 and 5, meeting the standard; CFI = 0.934 (greater than 0.90), SRMR = 0.044 (less than 0.08), RMSEA = 0.066 (less than 0.08) (see Tables 3, 4). These results indicate that the model constructed in this study achieved a good fit with the data (Hu and Bentler, 1999).

**Table 3:** Model fit measures (*N* = 508).

Measure	Estimate	Threshold	Interpretation
CMIN	838.292		
DF	269.000		
CMIN/DF	3.116	Between 1 and 5	Good
CFI	0.940	>0.90	Good
SRMR	0.042	<0.08	Excellent
RMSEA	0.065	<0.08	Good

CMIN = chi-square; DF = degree of freedom; CFI = comparative fit index; SRMR = standardized root mean square residual; RMSEA = root mean square error of approximation.

**Table 4:** Model fit measures (*N* = 560).

Measure	Estimate	Threshold	Interpretation
CMIN	933.918		
DF	269.000		
CMIN/DF	3.472	Between 1 and 5	Good
CFI	0.934	>0.90	Good
SRMR	0.044	<0.08	Excellent
RMSEA	0.066	<0.08	Good

CMIN = chi-square; DF = degree of freedom; CFI = comparative fit index; SRMR = standardized root mean square residual; RMSEA = root mean square error of approximation.

### Construct validity

This study assessed the construct validity of the questionnaire based on convergent validity and discriminant validity. The items’ convergent validity was evaluated using three criteria: item reliability, composite reliability (CR), and average variance extracted (AVE).

According to the standards of convergent validity, the questionnaire used in this study has high reliability. First, based on Hair et al. (2006), item reliability should be greater than 0.25. As shown in Figures 2, 3, the item reliability values in this study range from 0.37 to 0.87, indicating that all scales have high item reliability. At the same time, as shown in Tables 1, 2, the CR values for each factor range from 0.893 to 0.945, exceeding the 0.70 standard proposed by Hair et al. (2010), which indicates high internal consistency among the items of each factor. In addition, all AVE values range from 0.573 to 0.738 (see Tables 1, 2), which also exceed the minimum threshold of 0.50 proposed by Hair et al. (2010), indicating that all constructs basically meet the standard for convergent validity.

In terms of discriminant validity, the AVE values of each construct are greater than the correlation values with other related constructs (see Tables 1, 2), which meets the criterion suggested by Fornell and Larcker (1981) that the square root of each construct’s AVE should be greater than the correlations among constructs. Therefore, this study has excellent discriminant validity.

### Correlation analysis

This study aimed to explore the relationships among parental emotional warmth, life satisfaction, psychological resilience, and college students’ career choice anxiety. Pearson product–moment correlation coefficients were used to show the correlations among the variables.

As shown in Table 5, in the father sample, emotional warmth was significantly positively correlated with life satisfaction (*r =* 0.370, *p <* 0.001) and psychological resilience (*r =* 0.391, *p <* 0.001), and significantly negatively correlated with career choice anxiety (*r =* −0.090, *p =* 0.042). Life satisfaction was significantly positively correlated with psychological resilience (*r =* 0.614, *p <* 0.001), and significantly negatively correlated with career choice anxiety (*r =* −0.213, *p <* 0.001). Psychological resilience was significantly negatively correlated with career choice anxiety (*r =* −0.215, *p <* 0.001).

**Table 5:** Descriptive statistics and intercorrelations for study variables (*N* = 508/560).

Measure	*M*	*SD*	1	2	3	4
1. Pwarmth	4.038 (4.242)	1.092 (1.021)	1	0.414***	0.353***	−0.100*
2. Psychology	5.028 (5.029)	1.102 (1.080)	0.391***	1	0.619***	−0.260***
3. Satisfaction	4.304 (4.304)	1.252 (1.231)	0.370***	0.614***	1	**−** 0 .245***
4. Anxiety	4.875 (4.879)	1.372 (1.342)	−0.090*	−0.215***	−0.213***	1

Pwarmth = parental emotional warmth; Satisfaction = life satisfaction; Psychology = psychological resilience; Anxiety = career choice anxiety. The M and SD in parentheses are the mean and standard deviation, respectively, for the maternal sample. The bolded values above the diagonal are the Pearson product–moment correlation coefficients for the maternal sample.

***p < 0.001; **p < 0.01; *p < 0.05.

In the mother sample, emotional warmth was significantly positively correlated with life satisfaction (*r =* 0.353, *p =* 0.001) and psychological resilience (*r =* 0.414, *p <* 0.001), and significantly negatively correlated with career choice anxiety (*r =* −0.100, *p =* 0.018). Life satisfaction was significantly positively correlated with psychological resilience (*r =* 0.619, *p <* 0.001), and significantly negatively correlated with career choice anxiety (*r =* −0.245, *p <* 0.001). Psychological resilience was significantly negatively correlated with career choice anxiety (*r =* −0.260, *p <* 0.001).

### Path and mediation analysis

**Figure 4:**
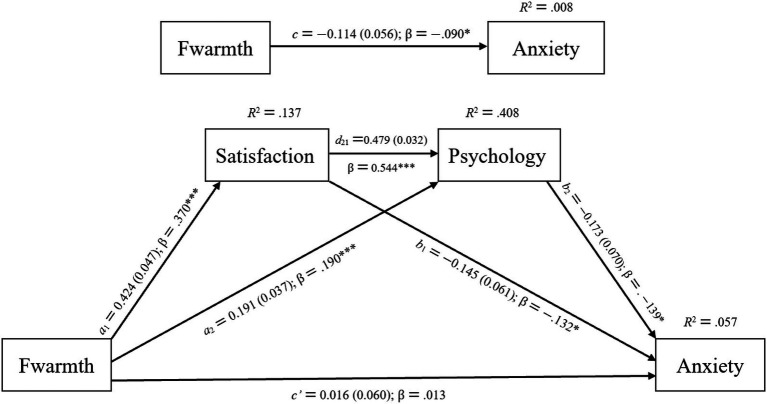
Model summary information for the hypothesized mediation model portrayed in Figure 1 (*N =* 508). Fwarmth = father’s emotional warmth; Satisfaction = life satisfaction; Psychology = psychological resilience; Anxiety = career choice anxiety. Standardized coefficients (β) and unstandardized regression coefficients *a*_1_, *a*_2_, *b*_1_, *b*_2_, *c*, *c*′, and *d*_21_ are presented along with their standard errors (shown in parentheses). *p < 0.05, **p < 0.01, ***p < 0.001.

**Figure 5:**
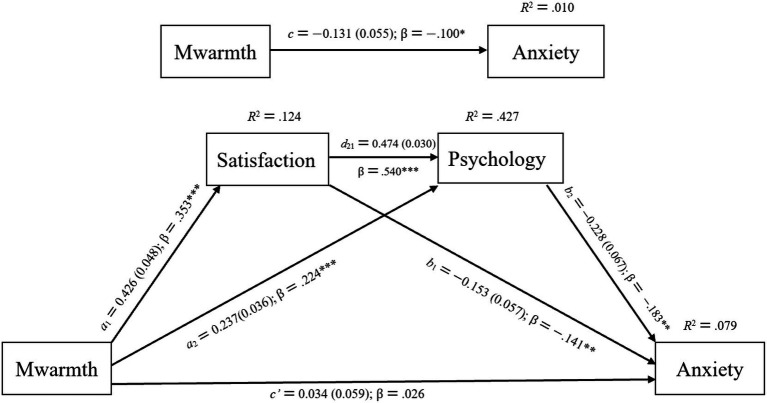
Model summary information for the hypothesized mediation model portrayed in Figure 1 (*N =* 560). Mwarmth = mother’s emotional warmth; Satisfaction = life satisfaction; Psychology = psychological resilience; Anxiety = career choice anxiety. Standardized coefficients (β) and unstandardized regression coefficients *a*_1_, *a*_2_, *b*_1_, *b*_2_, *c*, *c*′, and *d*_21_ are presented along with their standard errors (shown in parentheses). *p < 0.05, **p < 0.01, ***p < 0.001.

The results of the path analysis showed that parental emotional warmth was significantly negatively associated with college students’ career choice anxiety. In the father sample: *c = −*0.114, *SE =* 0.056, *β =* −0.090, *p <* 0.05, *R^2^ =* 0.008, *Adjusted R^2^ =* 0.006; in the mother sample: *c = −*0.131, *SE =* 0.055, *β =* −0.100, *p <* 0.05, *R^2^ =* 0.010, *Adjusted R^2^ =* 0.008 (see Figures 4, 5). After introducing life satisfaction and psychological resilience as mediating variables, the standardized regression coefficients of parental emotional warmth on career choice anxiety decreased and were no longer significant, which was statistically consistent with full mediation (Hayes, 2013). In the father sample: *c′ =* 0.016, *SE =* 0.060, *β =* 0.013, *p =* 0.790; in the mother sample: *c′ =* 0.034, *SE =* 0.059, *β =* 0.026, *p =* 0.569 (see Figures 4, 5; Tables 6, 7).

**Table 6:** Model summary information for the proposed mediation model portrayed in Figure 1 (*N* = 508).

Outcome									
Predictor	Satisfaction (Mediator 1)			Psychology (Mediator 2)			Anxiety		
	*B* (β)	*SE*	*p*	*B* (β)	*SE*	*p*	*B* (β)	*SE*	*p*
Fwarmth	*a_1_*→0.424 (0.370)	0.047	<0.001	*a_2_*→0.191 (0.190)	0.037	<0.001	*c′*→0.016 (0.013)	0.060	0.790
Satisfaction				*d_21_*→0.479 (0.544)	0.032	<0.001	*b_1_*→−0.145 (−0.132)	0.061	0.018
Psychology							*b*→−0.173 (−0.139)	0.070	0.014
Constant	2.592	0.198	<0.001	2.194	0.167	<0.001	6.302	0.305	<0.001
	*R*^2^ = 0.137 Adjusted *R*^2^ = 0.135 *F*(1, 506) = 80.175 *p <* 0.001			*R*^2^ = 0.408 Adjusted *R*^2^ = 0.406 *F*(2, 505) = 174.197 *p <* 0.001			*R*^2^ = 0.057 Adjusted *R*^2^ = 0.051 *F*(3, 504) = 10.105 *p <* 0.001		

Fwarmth = father’s emotional warmth; Satisfaction = life satisfaction; Psychology = psychological resilience; Anxiety = career choice anxiety. B = unstandardized regression coefficient; β = standardized regression coefficient.

**Table 7:** Model summary information for the proposed mediation model portrayed in Figure 1 (*N* = 560).

Outcome									
Predictor	Satisfaction (Mediator 1)			Psychology (Mediator 2)			Anxiety		
	*B* (β)	*SE*	*p*	*B* (β)	*SE*	*p*	*B* (β)	*SE*	*p*
Mwarmth	*a_1_*→0.426 (0.353)	0.048	<0.001	*a_2_*→0.237 (0.224)	0.036	<0.001	*c′*→0.034 (0.026)	0.059	0.569
Satisfaction				*d_21_*→0.474 (0.540)	0.030	<0.001	*b_1_*→−0.153 (−0.141)	0.057	0.007
Psychology							*b_2_*→−0.228 (−0.183)	0.067	0.001
Constant	2.498	0.208	<0.001	1.986	0.166	<0.001	6.541	0.293	*<*0.001
	*R*^2^ = 0.124 Adjusted *R*^2^ = 0.123 *F*(1, 558) = 79.297 *p <* 0.001			*R*^2^ = 0.427 Adjusted *R*^2^ = 0.425 *F*(2, 557) = 207.599 *p <* 0.001			*R*^2^ = 0.079 Adjusted *R*^2^ = 0.075 *F*(3, 556) = 16.006 *p <* 0.001		

Fwarmth = father’s emotional warmth; Satisfaction = life satisfaction; Psychology = psychological resilience; Anxiety = career choice anxiety. B = unstandardized regression coefficient; β = standardized regression coefficient.

Parental emotional warmth was significantly associated with life satisfaction. In the father sample: *a_1_ =* 0.424, *SE =* 0.047, *β =* 0.370, *p <* 0.001, *R^2^* = 0.137, *Adjusted R^2^ =* 0.135; in the mother sample: *a*_1_ *=* 0.426, *SE =* 0.048, *β =* 0.353, *p <* 0.001, *R^2^* = 0.124, *Adjusted R^2^ =* 0.123 (see Figures 4, 5; Tables 6, 7). Life satisfaction was significantly associated with career choice anxiety. In the father sample: *b_1_ = −*0.145, *SE =* 0.061, *β = −*0.132, *p =* 0.018; in the mother sample: *b_1_ = −*0.153, *SE =* 0.057, *β = −*0.141, *p =* 0.007 (see Figures 4, 5; Tables 6, 7). This study used the bootstrap method provided by the PROCESS macro for SPSS, with 10,000 resamples to test the mediation effect (Hayes, 2013). The results showed that in the father sample, *a*_1_×*b*_1_ = −0.061, with a 95% bootstrap confidence interval (CI) of [−0.121, −0.007]; in the mother sample, *a*_1_×*b*_1_ = −0.065, with a 95% bootstrap CI of [−0.129, −0.012]. Since the 95% bootstrap CI of the mediation effect did not include 0, it indicates that the mediation effect reached a significant level (see Tables 8, 9). Therefore, Hypothesis H1 was supported.

**Table 8:** Summary of the mediation model analysis (*N* = 508).

Effect type	Mediator	Equation	Point estimate	*SE*	95% bootstrap CI	
					*LL*	*UL*
Indirect effect 1	Satisfaction	*a*_1_ × *b*_1_	−0.061	0.029	−0.121	−0.007
Indirect effect 2	Psychology	*a*_2_ × *b*_2_	−0.033	0.018	−0.074	−0.001
Indirect effect 3	Satisfaction + Psychology	*a*_1_ × *d*_21_ × *b*_2_	−0.035	0.018	−0.071	−0.002
Total indirect effect		*a*_1_*b*_1_ + *a*_2_*b*_2_ + *a*_1_*d*_21_*b*_2_	−0.130	0.037	−0.203	−0.061
				*SE*	95% CI	
					*LL*	*UL*
Direct effect		*c′*	0.016	0.060	−0.102	0.134
Total effect		*c* = *c′* + *a*_1_*b*_1_ + *a*_2_*b*_2_ + *a*_1_*d*_21_*b*_2_	−0.114	0.056	−0.223	−0.004

Satisfaction = life satisfaction; Psychology = psychological resilience; Anxiety = career choice anxiety. CI = confidence interval; LL = lower limit; UL = upper limit. If the confidence interval does not include zero, it reveals a significant effect.

**Table 9:** Summary of the mediation model analysis (*N* = 560).

Effect type	Mediator	Equation	Point estimate	*SE*	95% bootstrap CI	
					*LL*	*UL*
Indirect effect 1	Satisfaction	*a*_1_ × *b*_1_	−0.065	0.030	−0.129	−0.012
Indirect effect 2	Psychology	*a*_2_ × *b*_2_	−0.054	0.021	−0.100	−0.017
Indirect effect 3	Satisfaction + Psychology	*a*_1_ × *d*_21_ × *b*_2_	−0.046	0.017	−0.081	−0.015
Total indirect effect		*a*_1_*b*_1_ + *a*_2_*b*_2_ + *a*_1_*d*_21_*b*_2_	−0.165	0.036	−0.239	−0.100
				*SE*	95% CI	
					*LL*	*UL*
Direct effect		*c′*	0.034	0.059	−0.083	0.150
Total effect		*c* = *c′* + *a*_1_*b*_1_ + *a*_2_*b*_2_ + *a*_1_*d*_21_*b*_2_	−0.131	0.055	−0.240	−0.023

Satisfaction = life satisfaction; Psychology = psychological resilience; Anxiety = career choice anxiety. CI = confidence interval; LL = lower limit; UL = upper limit. If the confidence interval does not include zero, it reveals a significant effect.

Parental emotional warmth was significantly associated with psychological resilience. In the father sample: *a_2_* = 0.191, *SE* = 0.037, *β = −*0.190, *p <* 0.001; in the mother sample: *a_2_* = 0.237, *SE* = 0.036, *β = −*0.224, *p <* 0.001 (see Figures 4, 5; Tables 6, 7). Psychological resilience was significantly associated with career choice anxiety. In the father sample: *b*_2_ = *−*0.173, *SE* = 0.070, *β = −*0.139, *p* = 0.014; in the mother sample: *b*_2_ = *−*0.228, *SE* = 0.067, *β = −*0.183, *p* = 0.001. Using the bootstrap method with 10,000 resamples, the results showed that in the father sample, *a*_2_ × *b*_2_ *= −*0.033, with a 95% bootstrap CI of [*−*0.074, *−*0.001]; in the mother sample, *a*_2_ × *b*_2_ *= −*0.054, with a 95% bootstrap CI of [*−*0.100, *−*0.017]. Since the 95% bootstrap CI of the mediation effect did not include 0, it indicates that the mediation effect reached a significant level (see Tables 8, 9). Therefore, Hypothesis H2 was supported.

In addition, the effect of life satisfaction on psychological resilience was also significant. In the father sample: *d_21_ =* 0.479, *SE =* 0.032, *β =* 0.544, *p <* 0.001; in the mother sample: *d_21_ =* 0.474, *SE =* 0.030, *β =* 0.540, *p <* 0.001. When life satisfaction and psychological resilience were taken as chain mediators, in the father sample: *a_1_* × *d_21_* × *b_2_ = −*0.035, 95% bootstrap CI *=* [−0.071, −0.002]; in the mother sample: *a_1_* × *d_21_* × *b_2_ = −*0.046, 95% bootstrap CI *=* [−0.081, −0.015] (see Tables 8, 9). Since the 95% bootstrap CI of the mediation effect did not include 0, it indicates that the chain mediation effect reached a significant level. Taken together, this mediation effect was statistically consistent with full mediation. Therefore, Hypothesis H3 was supported.

## Discussion

### Summary of key findings

This study examined the relationship between parental emotional warmth and career choice anxiety among Chinese college students, with particular attention to the chain-mediating roles of life satisfaction and psychological resilience. The results showed that emotional warmth from both fathers and mothers was associated with lower career choice anxiety at the overall level, but this association became indirect once life satisfaction and psychological resilience were included in the model. In other words, the effect of parental emotional warmth on career choice anxiety was statistically consistent with full mediation by these two psychological resources. This finding suggests that parental emotional warmth may not be directly linked to career-related distress; but rather as an important family context that fosters positive internal resources, which in turn may be associated with lower anxiety during the career decision-making process. These findings extend existing research on family influences and youth anxiety by clarifying how supportive parenting may shape career-related emotional adjustment in the Chinese higher education context.

### Comparison with previous research

Consistent with Self-Determination Theory (Ryan and Deci, 2000) and Ecological Systems Theory (Bronfenbrenner, 1986), the findings support the view that emotionally warm parenting provides a supportive developmental environment in which students are more likely to cultivate adaptive psychological resources. More specifically, the full mediation pattern suggests that parental emotional warmth may function primarily as a distal contextual influence, whereas life satisfaction and psychological resilience serve as more proximal psychological mechanisms that are more directly associated with career choice anxiety. From this perspective, emotionally warm parenting may not reduce career anxiety in a direct and immediate manner; instead, it may first enhance students’ overall evaluation of life and their ability to cope with challenges, which then may be linked to lower anxiety when they face uncertain or demanding career decisions.

This interpretation is theoretically meaningful because career choice anxiety is often shaped by students’ immediate perceptions of uncertainty, coping capacity, and future controllability, rather than by family climate alone. Thus, once life satisfaction and psychological resilience are taken into account, the direct effect of parental emotional warmth may become negligible because its influence is already transmitted through these more proximal psychological pathways. At the same time, this full mediation pattern should be interpreted cautiously. It would be premature to conclude that parental emotional warmth affects career choice anxiety only through life satisfaction and psychological resilience, as the cross-sectional design and reliance on self-report measures may also have attenuated the direct effect. Therefore, the present findings are better understood as suggesting that, in this sample, the association between parental emotional warmth and career choice anxiety operated primarily through these internal psychological resources.

The present findings also support prior research showing that life satisfaction functions as an important cognitive-emotional buffer against stress (Diener et al., 2018; Müceldili et al., 2023), while psychological resilience promotes adaptive functioning in the face of pressure and uncertainty (Luthar et al., 2000; Southwick and Charney, 2012). In this sense, students who perceive greater emotional warmth from their parents may develop a more positive orientation toward life, which then strengthens their resilience and may be associated with reduced vulnerability to career-related anxiety. However, our findings differ from some Western studies suggesting that resilience may occasionally be associated with overthinking or decision paralysis under highly individualized decision-making conditions (Kenner and Sage, 2023). One possible explanation is that, in the Chinese cultural context, resilience may be more closely linked to perseverance, relational support, and adaptive endurance, making it more consistently protective in the face of career uncertainty.

A further culturally relevant issue concerns the potential “warmth–pressure” paradox in Chinese families. Although high parental involvement may sometimes become a source of pressure when expectations exceed children’s perceived abilities, such a paradoxical effect was not observed in the present study. One possible explanation is that the parental emotional warmth measure primarily captured affective support, care, and responsiveness rather than expectation-based pressure or psychologically controlling forms of involvement. In addition, because life satisfaction and psychological resilience functioned as protective mediators in the present study, it is possible that parental warmth was more likely to operate through supportive psychological pathways than through pressure-related mechanisms in this sample. However, this interpretation should be treated cautiously, as the present study did not directly assess students’ subjective interpretations of parental involvement or expectation-based pressure. Future research may therefore benefit from distinguishing emotional warmth from expectation-laden parental involvement more explicitly.

Furthermore, whereas some Western studies have reported stronger maternal influence in emotional development (e.g., Stocker et al., 2016), Our results showed no substantial difference between paternal and maternal emotional warmth in their indirect associations with career choice anxiety through the mediators. This finding aligns more closely with research in East Asian contexts (Rudy and Grusec, 2006), where both fathers and mothers are often actively involved in children’s long-term educational and developmental planning. This pattern may also be interpreted in light of the culturally relative meaning of emotional warmth in Chinese families. As noted earlier, Chinese parents often express care not only through affection and responsiveness but also through encouraging expectations regarding children’s future development. In this cultural context, such support-oriented involvement is not limited to mothers; fathers may also communicate concern, responsibility, and developmental expectations in ways that are experienced as emotional investment. In the Chinese higher education context, career choice is often perceived as a family-related developmental task rather than a purely individual decision. Accordingly, emotional warmth from both parents may contribute in comparable ways to the development of life satisfaction and resilience, thereby being indirectly associated with lower career choice anxiety. Taken together, these findings help address a cultural gap in the literature by showing that the family–career anxiety linkage may operate through similar psychological mechanisms across both paternal and maternal pathways in collectivist settings.

### Theoretical and practical implications

#### Theoretical implications

This study contributes to theoretical advancement in several ways. First, it establishes a chain mediation model in which cognitive well-being (life satisfaction) and emotional adaptability (resilience) were statistically consistent with explaining how parental warmth was associated with career choice anxiety—an integrated mechanism rarely examined in tandem. Second, it refines existing models of career psychological adaptation by emphasizing the indirect and context-dependent pathways through which family support may be associated with stress responses. Third, the inclusion of both maternal and paternal influences in a non-Western setting extends cross-cultural applications of family systems theory and positive psychology.

#### Practical implications

From a practical standpoint, the findings underscore the foundational role of family emotional support in understanding and potentially alleviating college students’ career stress. Universities should strengthen parent–school collaboration by offering workshops or counseling services that help families differentiate between supportive warmth and performance pressure. At the institutional level, student support centers should prioritize interventions that promote life satisfaction and resilience, such as mindfulness programs, peer support systems, and adversity training modules. Additionally, early screening for students with low satisfaction or poor coping should be embedded in career services to enable tiered psychological support.

### Limitations and future research

Despite these contributions, several limitations should be acknowledged. First, the study used cross-sectional data from universities in Southeastern China, which limits causal inference and broader generalizability. In addition, participants were recruited through purposive sampling via online forums and WeChat groups, which may have introduced selection bias. Students who are more active on digital platforms or more willing to complete online surveys may have been overrepresented, and such respondents may differ from non-participants in career choice anxiety, perceived parental warmth, or psychological adjustment. Accordingly, the findings should be generalized with caution. Future research should use more diverse recruitment channels or probability-based sampling methods to improve sample representativeness. Future research should also employ longitudinal or experimental designs to test directional relationships more rigorously.

Second, although validated measures were used, some scales were abridged or adapted, which may have affected cross-cultural equivalence. Although item removal was theoretically and statistically justified, the shortened versions of the career choice anxiety scale and the psychological resilience scale should be further cross-validated in future research. In addition, because all variables were self-reported, shared method variance may still have influenced the observed associations. Mixed-method approaches could help triangulate the findings and improve ecological validity.

Third, the study did not examine potential moderators such as personality traits or cultural values (e.g., perfectionism and collectivism), which may shape how parental warmth relates to career choice anxiety. Future studies should investigate these conditional mechanisms across diverse sociocultural groups. Finally, although this study focused on Chinese college students, comparative research involving other East Asian and Western populations would help clarify which findings are culture-specific and which may be more universal.

## Conclusion

Situated within the Chinese higher education context, this study underscores the culturally specific role of parental emotional warmth in relation to career choice anxiety. In contrast to Western individualist models, where autonomy may dominate career development, this study highlights the significance of collectivist family dynamics—particularly emotional support from both parents—in shaping students’ psychological resilience and life satisfaction during career transitions.

By constructing and validating a chain-mediation model, the study reveals that parental emotional warmth was indirectly associated with two critical psychological resources: life satisfaction and psychological resilience. When both mediators are included, the direct effect becomes non-significant, which is statistically consistent with full mediation. This finding offers nuanced insight into how cognitive and emotional mechanisms interact to be associated with lower anxiety in high-pressure decision contexts.

Theoretically, the study advances understanding in the fields of career psychology, positive youth development, and stress management by integrating self-determination theory and ecological systems theory. Practically, it underscores the equal importance of both fathers’ and mothers’ warmth in Chinese families—challenging assumptions of maternal primacy in emotional socialization. These results provide actionable implications for educational institutions and policymakers striving to alleviate career anxiety and the growing “slow employment” phenomenon through family-school-community collaboration.

## Data Availability

The datasets presented in this study can be found in online repositories. The names of the repository/repositories and accession number(s) can be found in the article/Supplementary material.
