# Supplementary material for: Parental emotional warmth and career choice anxiety: a chain-mediation study in China
Source: Front Psychol. 2026 May 21;17:1813789. doi: 10.3389/fpsyg.2026.1813789 (PMC13233494; doi:10.3389/fpsyg.2026.1813789)
Supplement: Supplementary file 1 [file Supplementary_File_1.pdf]

Supplementary Materials

Measurement Items and Standardized Factor Loadings Before and After Item Removal

Note. All items were administered in Chinese. The original Chinese wording is reported below to reflect the exact measurement content used in the present study. Standardized factor loadings were obtained from confirmatory factor analysis (CFA). "Original model" refers to the initially specified measurement model before item removal. "Final model" refers to the respecified model after item removal. Items marked as "Deleted" were removed during CFA model respecification due to comparatively weaker psychometric performance and/or poorer conceptual alignment with the focal construct in the present study. Dashes (—) indicate that the item was not retained in the final model.

Table S1

Measurement Items and Standardized Factor Loadings for the Adapted Career Choice Anxiety Scale (Father Sample)

Item code	Item wording (Chinese)	Original model loading	Final model loading	Status	Brief rationale
CCA1	想到毕业要选择职业，我无缘无故感到担心。	.81	.82	Retained	—
CCA2	想到毕业要选择职业，我感到比平时更加紧张和焦虑。	.85	.86	Retained	—
CCA3	想到毕业要选择职业，我容易心里烦乱或觉得惊恐。	.94	.94	Retained	—
CCA4	想到毕业要选择职业，我感到虚弱且容易疲乏。	.82	.81	Retained	—
CCA5	想到毕业要选择职业，我感觉我的心跳较快。	.42	—	Deleted	comparatively weaker loading and less direct relevance to career choice anxiety in the present context

Note. CCA = Career Choice Anxiety. The retained items continued to capture the core affective features of career choice anxiety in the present study.

Table S2

Measurement Items and Standardized Factor Loadings for the Adapted Career Choice Anxiety Scale (Mother Sample)

Item code	Item wording (Chinese)	Original model loading	Final model loading	Status	Brief rationale
CCA1	想到毕业要选择职业，我无缘无故感到担心。	.80	.81	Retained	—
CCA2	想到毕业要选择职业，我感到比平时更加紧张和焦虑。	.85	.86	Retained	—
CCA3	想到毕业要选择职业，我容易心里烦乱或觉得惊恐。	.94	.92	Retained	—
CCA4	想到毕业要选择职业，我感到虚弱且容易疲乏。	.81	.79	Retained	—
CCA5	想到毕业要选择职业，我感觉我的心跳较快。	.43	—	Deleted	comparatively weaker loading and less direct relevance to career choice anxiety in the present context

Note. CCA = Career Choice Anxiety. The retained items continued to capture the core affective features of career choice anxiety in the present study.

Table S3

Measurement Items and Standardized Factor Loadings for the Psychological Resilience Scale (Father Sample)

Item code	Item wording (Chinese)	Original model loading	Final model loading	Status	Brief rationale
PR1	当发生变化时，我能灵活适应。	.85	.85	Retained	—
PR2	遇到困难时，我都可以处理。	.83	.83	Retained	—
PR3	面对问题时，我会幽默应对。	.83	.83	Retained	—
PR4	通过经验的积累，使我变得更加坚强。	.43	—	Deleted	comparatively weaker loading and poorer contribution to construct coherence in the present sample
PR5	经历生病或苦难后，我的复原能力很强。	.77	.77	Retained	—
PR6	即使遇到障碍，我也可以实现目标。	.86	.86	Retained	—
PR7	面临压力时，我能聚精会神思考问题。	.80	.80	Retained	—
PR8	我不会因失败而沮丧。	.74	.74	Retained	—
PR9	面临生活中挑战时，我认为我是个坚强的人。	.85	.86	Retained	—
PR10	我有能力处理不愉快的感觉，例如生气。	.76	.77	Retained	—

Note. PR = Psychological Resilience. The retained items continued to represent the core conceptual domain of psychological resilience in the present study.

Table S4

Measurement Items and Standardized Factor Loadings for the Psychological Resilience Scale (Mother Sample)

Item code	Item wording (Chinese)	Original model loading	Final model loading	Status	Brief rationale
PR1	当发生变化时，我能灵活适应。	.83	.83	Retained	—
PR2	遇到困难时，我都可以处理。	.81	.81	Retained	—
PR3	面对问题时，我会幽默应对。	.81	.81	Retained	—
PR4	通过经验的积累，使我变得更加坚强。	.47	—	Deleted	comparatively weaker loading and poorer contribution to construct coherence in the present sample
PR5	经历生病或苦难后，我的复原能力很强。	.76	.76	Retained	—
PR6	即使遇到障碍，我也可以实现目标。	.87	.87	Retained	—
PR7	面临压力时，我能聚精会神思考问题。	.79	.79	Retained	—
PR8	我不会因失败而沮丧。	.73	.73	Retained	—
PR9	面临生活中挑战时，我认为我是个坚强的人。	.84	.84	Retained	—
PR10	我有能力处理不愉快的感觉，例如生气。	.76	.76	Retained	—

Note. PR = Psychological Resilience. The retained items continued to represent the core conceptual domain of psychological resilience in the present study.

Figure S1

The Original CFA Model Summary Information Prior to Item Removal (Father Sample)

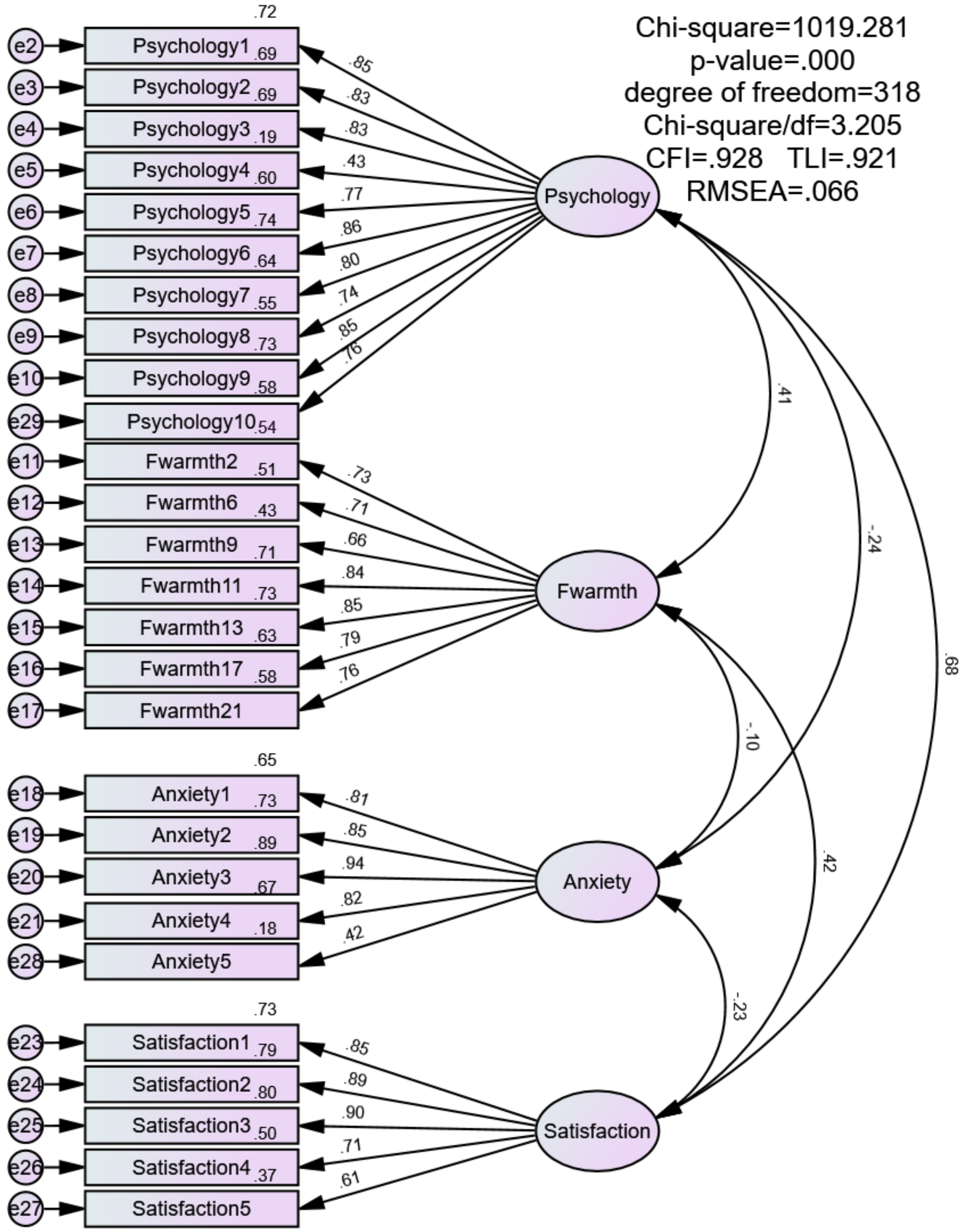

Note. Fwarmth = Father's Emotional Warmth; Satisfaction = Life Satisfaction; Psychology = Psychological Resilience; Anxiety = Career Choice Anxiety. This figure presents the initially specified CFA model before item removal.

Figure S2

The Original CFA Model Summary Information Prior to Item Removal (Mother Sample)

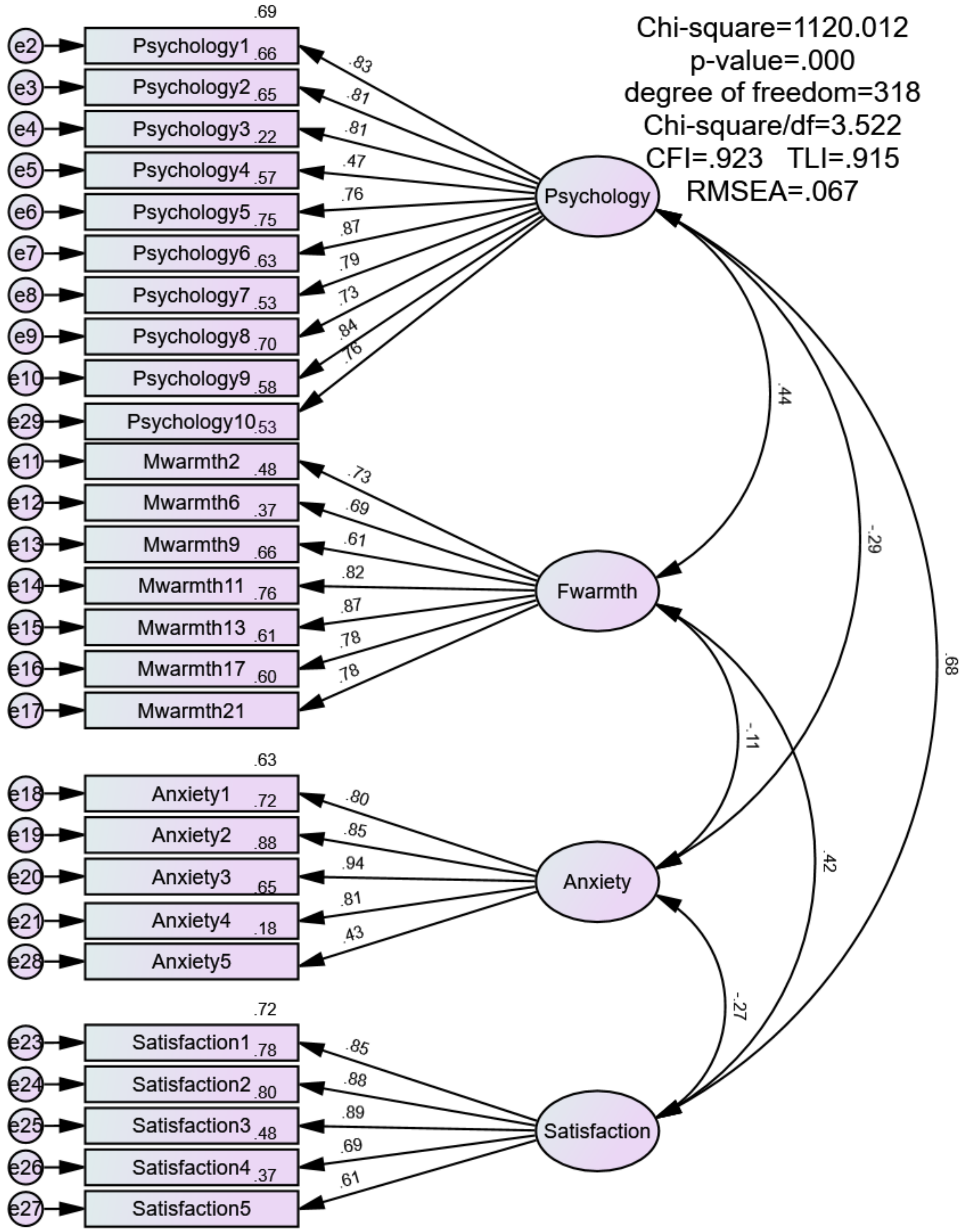

Note. Mwarmth = Mother's Emotional Warmth; Satisfaction = Life Satisfaction; Psychology = Psychological Resilience; Anxiety = Career Choice Anxiety. This figure presents the initially specified CFA model before item removal.
